# The Stereochemistry of Complex Polyketide Biosynthesis by Modular Polyketide Synthases

**DOI:** 10.3390/molecules16076092

**Published:** 2011-07-20

**Authors:** David H. Kwan, Frank Schulz

**Affiliations:** 1 Department of Chemistry, University of British Columbia, 2036 Main Mall, Vancouver B.C., V6T 1Z1, Canada; 2 Fakultät für Chemie, Chemische Biologie, Technische Universität Dortmund, Otto-Hahn-Straße 6, 44221 Dortmund, Germany; 3 Max-Planck-Institut für Molekulare Physiologie, Abteilung für Chemische Biologie, Otto-Hahn- Straße 11, 44227 Dortmund, Germany

**Keywords:** polyketides, polyketide synthase, stereochemistry, ketosynthase, acyltransferase, ketoreductase, dehydratase, enoylreductase

## Abstract

Polyketides are a diverse class of medically important natural products whose biosynthesis is catalysed by polyketide synthases (PKSs), in a fashion highly analogous to fatty acid biosynthesis. In modular PKSs, the polyketide chain is assembled by the successive condensation of activated carboxylic acid-derived units, where chain extension occurs with the intermediates remaining covalently bound to the enzyme, with the growing polyketide tethered to an acyl carrier domain (ACP). Carboxylated acyl-CoA precursors serve as activated donors that are selected by the acyltransferase domain (AT) providing extender units that are added to the growing chain by condensation catalysed by the ketosynthase domain (KS). The action of ketoreductase (KR), dehydratase (DH), and enoylreductase (ER) activities can result in unreduced, partially reduced, or fully reduced centres within the polyketide chain depending on which of these enzymes are present and active. The PKS-catalysed assembly process generates stereochemical diversity, because carbon–carbon double bonds may have either *cis*- or *trans*- geometry, and because of the chirality of centres bearing hydroxyl groups (where they are retained) and branching methyl groups (the latter arising from use of propionate extender units). This review shall cover the studies that have determined the stereochemistry in many of the reactions involved in polyketide biosynthesis by modular PKSs.

## 1. Complex Polyketides

**Figure 1:**
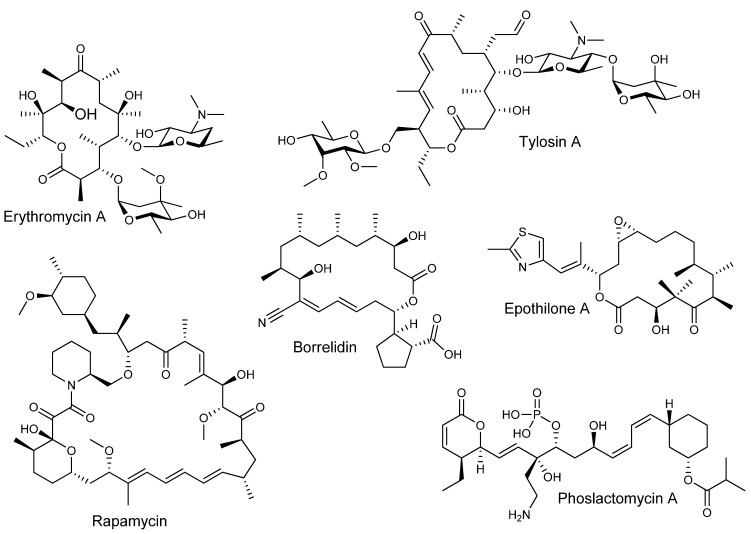
Bioactive complex polyketides with diverse structure and function.

Polyketides are among the most important microbial natural products used in medicine. Members of this diverse family of compounds are used as a wide variety of therapeutics, including antibiotics such as erythromycin, anticancer epothilones, immunosuppressant rapamycin, and cholesterol-lowering lovastatin, to name only a few [1,2,3,4]. This colourful spectrum of biological activities arises from their considerable structural diversity (Figure 1).

Polyketides are biosynthesized by plants, fungi, and bacteria from the step-wise condensation of short chain carboxylic acid-derived units. Assembly of complex structures from these simple building blocks is carried out by enzyme complexes known as polyketide synthases (PKSs). Different families of PKSs generate very distinct classes of polyketides. Aromatic polyketides, arising largely from unreduced polyketone chains, comprise one such class. These are distinct from the reduced or complex polyketides, in which many of the carbonyl functionalities have undergone partial or full reduction during biosynthesis by the action of component enzyme activities within the PKS. In contrast to aromatic polyketides, complex polyketides are biosynthesized by the catalytic action of unusually large (some ranging in megadaltons) multidomain enzymes forming a sort of molecular assembly-line wherein each catalytic step is carried out by a specific domain. The “assembly-line” PKS enzymes that biosynthesize complex polyketides are known as modular PKSs and contain catalytic domains that are usually covalently linked with the domains organized into “modules” in which the enzyme activities are specific to a given step in chain elongation. In what is described as the “co-linearity rule,” the organization of modules in modular PKSs and the catalytic activities present in each module ultimately determines the structure of the polyketide chain (with potential post-PKS tailoring or decoration of the chain occurring after polyketide biosynthesis). It is the modularity of these biosynthetic enzymes and the countless ways in which the “assembly-line” can be configured in modular PKSs of different organisms that gives rise to the vast structural and functional diversity of complex polyketides [5,6].

## 2. Modular Type I PKSs and the Closely Related Metazoan Type I FASs

The biosynthesis of polyketides shares a great deal of similarity to that of fatty acids, and much of our current understanding of PKS enzymes has benefited from extensive studies on the fatty acid synthase (FAS) systems [1,3]. By the 1980s, it was clearly evident from genetic analyses of the two systems that they shared a common evolutionary history [7,8,9]. PKSs, like FASs, can be broadly classified as type I or type II, with multiple catalytic activities contained either within large, covalently bound multienzyme complexes, or on discrete, dissociated enzymes respectively (PKSs can also be categorized into a type III classification, which are among the simplest PKSs known [10] and which shall not be further discussed in this review). Modular PKSs fall under the type I PKS classification, and are closely related to the type I metazoan (animal) FASs.

**Figure 2:**
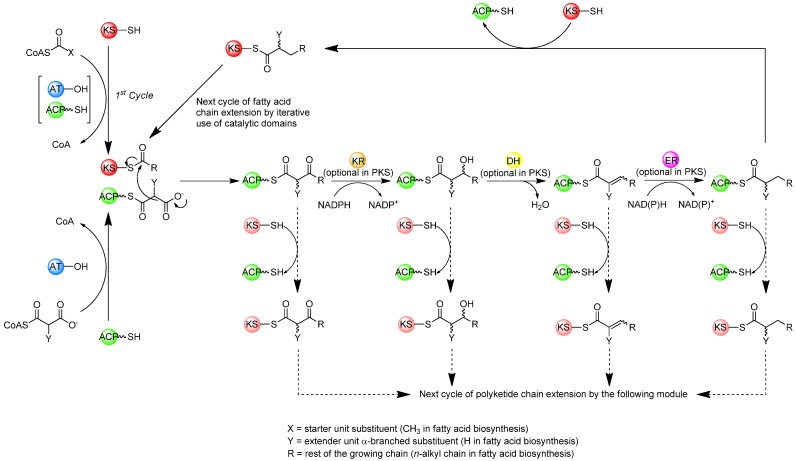
Biosynthesis of fatty acids (solid arrows) and polyketides (solid/dashed arrows).

In the sequence of reactions involved in the biosynthesis of both polyketides and fatty acids, beginning with a starter acyl group, extender units from activated acyl-CoA precursors are assembled head-to-tail into the growing chain in a highly co-ordinated fashion involving several enzymatic activities (Figure 2) [1,3]. The extender units are derived from acyl-CoA precursors activated by carboxylation at the α-position, allowing for chain elongation by decarboxylative condensation. In fatty acid biosynthesis the starter unit is an acetyl group derived from acetyl-CoA and the extender units are activated acetyl groups derived from malonyl-CoA, whereas polyketide biosynthesis can utilize a variety of starter units derived from acyl-CoA precursors and activated extender units derived from carboxylated acyl-CoA precursors. In both cases, the growing chain remains bound by thioester-linkage to the enzymes involved in extension. Typically, the process begins with the transfer of an acyl group to the ketosynthase (KS), an enzyme that catalyses condensation during elongation, where it is attached to a specific cysteine thiol. In animal FAS and modular PKS systems, the starter acyl unit is first transferred from the acyl-CoA by an acyltransferase (AT) to an acyl carrier protein (ACP), to which it is tethered *via* a phosphopantetheine cofactor attached in phosphodiester linkage to a unique serine residue, before being passed to the KS [11]. In the first elongation cycle, the AT loads the ACP with an extender unit. The KS then catalyses condensation of the ACP-tethered extender unit to the bound acyl group with concomitant decarboxylation of the carboxylated acyl moiety, resulting in an ACP-bound b-ketoacyl intermediate. The b-ketoacyl thioester intermediate can then be further modified at the b-carbon by a ketoreductase (KR – catalysing reduction of the keto group to a hydroxyl), a dehydratase (DH – catalysing dehydration of the hydroxyl to form a double bond), and an enoylreductase (ER – reducing the double bond to a saturated methylene). In FAS enzymes, the KR, DH, and ER domains are always present and active, reducing the C-3 position of the nascent chain to a saturated methylene, and the extended acyl chain is then passed again to the KS, for the next of a number of identical cycles in which the same enzymes catalyse iterative condensation with malonyl extender units, accompanied by b-carbon reduction of the intermediates until the chain is released by a thioesterase (TE). On the other hand, in modular PKSs, some of the KR, DH, and ER domains may or may not be present or active in a given ‘module’ where each successive chain extension is carried out by a different module containing its own set of catalytic domains necessary for chain extension (and optional b-carbon processing). While the end result of FAS-catalysed fatty acid biosynthesis is typically a fully reduced carbon chain of a defined length, polyketides resulting from assembly by modular PKSs usually have varying degrees of reduction at different carbon centres within the chain where the degree of reduction on the b-carbon-derived centres, and indeed the overall structure, of the polyketide depends on the presence or absence of these optional domains within the modules [1].

**Figure 3:**
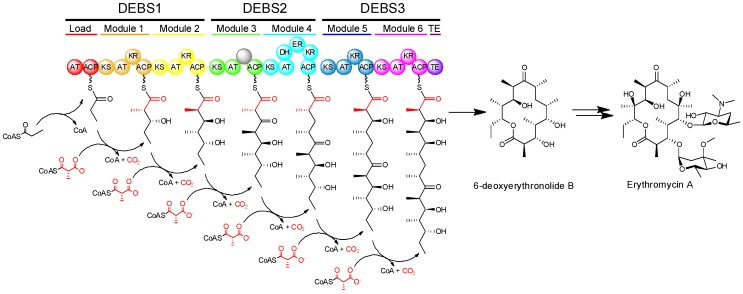
Biosynthesis of the aglycone of erythromycin by 6-deoxyerythronolide B synthase (DEBS) and subsequent conversion into erythromycin A.

The modular PKSs (at least those of actinomycete bacteria) typically exhibit what is called co-linearity with their products, that is to say that by examination of the sequence of modules of the PKS, one can make a reasonably accurate prediction about the structure of the resulting product [1]. One example showing how modular PKS organization is reflected in polyketide structure, can be seen in the multienzyme complex known as 6-deoxyerythronolide B synthase (DEBS), which synthesizes the aglycone core of erythromycin (Figure 3). DEBS is a modular PKS in which six extension modules in addition to a loading module, divided among three large proteins (DEBS1, DEBS2, and DEBS3), work in an assembly-line fashion to produce 6-deoxyerythronolide B. The DEBS system, which was the first modular PKS to be sequenced [12], remains the best studied to date.

The modular PKSs are versatile enzymes responsible for the biosynthesis of a very large number of diverse complex or reduced polyketides with varied structure. Erythromycin represents a sub-family of such polyketide compounds known as macrolides, which consist of large (12-, 14-, or 16-membered) macrocyclic lactone rings bearing unusual deoxy(amino)sugar moieties. The polyethers, which include the ionophoric antibiotic monensin, are formed by the oxidative cyclization of an initially synthesized linear polyketide polyene [13]. Other complex polyketides are hybrids of different structural classes; rapamycin is formed by the action of rapamycin synthase (RAPS), which includes type I PKS modules and also a non-ribosomal peptide synthetase (NRPS) module (another example of a modular assembly-line system), forming a mixed polyketide-peptide structure. Amongst this structural diversity, modular PKSs generate an impressive diversity of asymmetric centres in their polyketide products. This occurs as the reduction of b-ketones to alcohols gives rise to stereogenic centres bearing hydroxyl groups (where they are retained), branching methyl groups may be incorporated in different configurations, and the double bonds that are formed may have either *cis* or *trans* geometry. As with other aspects of polyketide biosynthesis, many of the early insights on the stereochemistry of reactions catalysed by PKSs were gained by analogy to the highly related FASs.

## 3. Stereochemical Aspects of Fatty Acid Biosynthesis

Although the products of the fatty acid biosynthetic pathway are typically achiral, pioneering studies have elucidated the entire stereochemical course of their assembly. Understanding of the detailed stereochemistry underlying the reaction mechanisms involved in fatty acid biosynthesis has led to important insights into how stereocentres are generated in polyketides.

**Figure 4:**
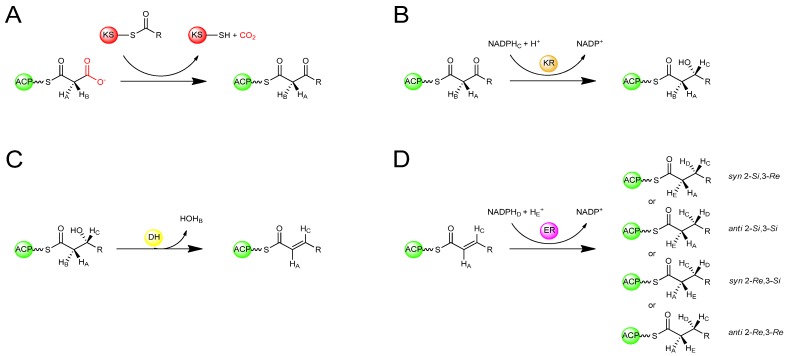
Stereochemical course of reactions during fatty acid biosynthesis [14,15,16,17,18,19,20,21]. (**A**) Stereochemical inversion at C-2 upon KS-catalysed condensation. (**B**) Ketoreduction of the 3-ketone to produce a 3*R*-configured b-hydroxyl intermediate. (**C**) Dehydration of the b-hydroxyl intermediate generating a *trans*-configured double-bond (**D**) Enoylreduction of the unsaturated intermediate in varying organisms by alternative stereochemical courses.

Stereospecific isotopic labelling of FAS substrates has enabled the otherwise cryptic stereospecificity of the enzymes to be revealed. By labelling prochiral centres of the substrates with deuterium or tritium and tracing the fate of the isotopic labels, the stereochemical course of each reaction has been determined (Figure 4). Using this approach, it was deduced that the KS-catalysed condensation reaction in the type I FASs of both animals and yeast occurs with inversion of configuration at the C-2 position (Figure 4A) [14,15]. The KR-catalysed reduction of the b-ketoacyl intermediate occurs by the attack on the *si* face of the C-3 carbonyl carbon by the 4′-*pro-S* hydride of NADPH to give exclusively the (3*R*)-hydroxyl intermediate (Figure 4B) [16]. This is followed by a DH-catalysed *syn* elimination of water from the 2-*pro-S* hydrogen and the (3*R*)-hydroxyl group generating a *trans* double bond (Figure 4C) [17]. The ER is the only enzyme of FAS known to differ in its stereochemical course among different organisms (Figure 4D). The ER of animal FAS, catalyses attack by the 4′-*pro*-*R* hydride of NADPH at the 3-*Re* face, with protonation at the 2-*Si* face, giving an overall *syn* addition [14]. The ER of yeast FAS catalyses attack by the 4′-*pro*-*S* hydride of NADPH [18] at the 3-*Si* face, with protonation occuring at the 2-*Si* face, an overall *anti* addition [19]. In the type II FAS of *E. coli*, the ER catalyses attack by the 4′-*pro*-*S* hydride of NADH at the 3-*Si* face of the enoyl intermediate with protonation at the 2-*Re* face [20] to give overall *syn* addition. It has also been discovered that in dyer’s thistle (*Carthamus tinctorius*) and flour beetle (*Tribolium confusumon*), both of which produce unusual hydrocarbons from fatty acids, an ER activity catalyses the *anti*-2-*Re*,3-*Re* addition in the biosynthesis of these compounds [21]. Thus, remarkably all four possible stereochemical courses for enoylreduction have been observed in nature among FAS enzymes from different organisms.

## 4. Stereochemical Control of Assembly-Line Biosynthesis in Modular Type I PKSs

The modular PKSs typically generate, within their complex polyketide products, a multitude of stereogenic centres. How the modular PKSs exert control over the configuration of each of these centres has been the subject of intensive study. Stereospecificity is observed in each step in the sequence of reactions in a given cycle of chain extension, controlling not only the configuration of the functionality at the b-carbon-derived centre, from the processing of the nascent b-keto group, but also the configuration at the a-carbon-derived centre in cases where a branched extender unit, such as a methylmalonyl unit, is introduced. 

**Figure 5:**
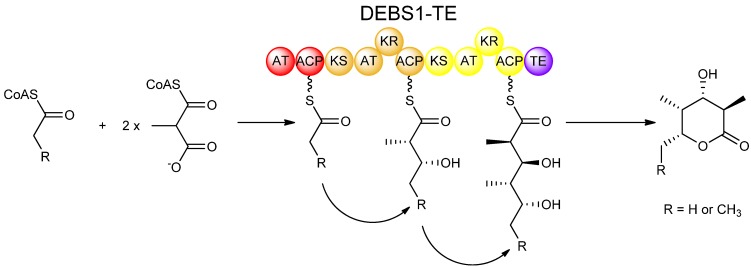
Synthesis of a triketide lactone by DEBS1-TE, a recombinant PKS engineered by fusing the TE domain of DEBS3 to the C-terminus of the DEBS1 protein [22].

As with fatty acid biosynthesis, isotopic labeling studies proved useful in determining the stereochemical course of reactions in polyketide biosynthesis. Expression of isolated PKS domains made it possible to study many of the catalytic activities individually *in vitro*. Also, the use of engineered recombinant hybrid PKSs greatly facilitated studies on many aspects of polyketide biosynthesis including stereochemistry. One such hybrid PKS is the bimodular PKS known as DEBS1-TE, which was engineered by relocating the chain-terminating thioesterase (TE) domain from DEBS3 and fusing it to the C-terminus of the DEBS1 protein, housing the loading module and first two extension modules [22]. The resulting DEBS1-TE, which produces a triketide lactone product (Figure 5), provides a relatively simple model PKS system, and has served as the work-horse for many studies on polyketide biosynthesis including experiments involving domain-swapping, site-directed mutagenesis, and *in vitro* feeding studies which have been invaluable in understanding the stereochemistry of PKS-catalysed reactions.

### 4.1. Stereoselectivity of the AT-Catalysed Acyl Transfer

The AT domain of modular PKSs is responsible for the recruitment of the extender units used in the chain elongation of polyketide biosynthesis. The AT catalyses the attachment of the selected extender unit to the ACP of a given module. The specificity for different extender units differs among AT domains, and divergent sequence motifs which correlate to extender unit specificity (e.g., malonyl or methylmalonyl) have been identified [23,24,25]. For the erythromycin biosynthetic pathway, which utilizes methylmalonyl units for each extension step, many studies using isotopically labeled precursors have been carried out and have determined that the AT domains of DEBS modules utilize solely the (2*S*)- isomer of methylmalonyl-CoA.

**Figure 6:**
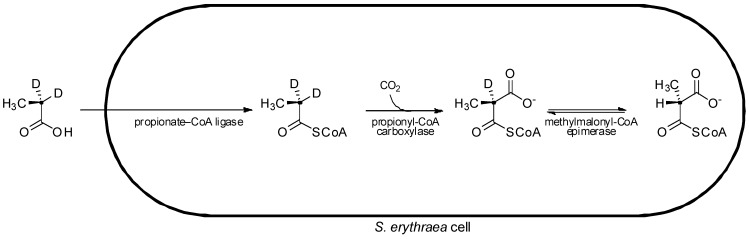
Feeding of labeled precursor, [2-^2^H_2_,2-^13^C]propionate, to *S. erythraea* and subsequent *in situ *intracellular conversion to (2*R*)-[2-^13^C]methylmalonyl-CoA and (2*S*)-[2-^2^H,2-^13^C]methylmalonyl-CoA [26].

In pioneering studies on the stereochemistry of erythromycin biosynthesis, cultures of erythromycin-producing *S. erythraea* were fed isotopically labeled [2-^2^H_2_,2-^13^C]propionate. From the labelled propionate, two stereoisomers of isotopically labelled methylmalonyl-CoA were generated intracellularly ((2*S*)-[2-^2^H,2-^13^C]methylmalonyl-CoA and (2*R*)-[2-^13^C]methylmalonyl-CoA), with the (2*S*)- isomer bearing a deuterium label (Figure 6) [26]. Although exchange of the deuterium occurring on the (2*S*)- precursor hampered its incorporation into the polyketide, analysis of the macrolide product resulting from assembly of the deuterated (2*S*)- extender units showed evidence of deuterium labeling at the C-2, C-4, and C-10 centres, where D-configured methyl substituents were present, indicating that the extender units incorporated at these positions had arisen from (2*S*)-methylmalonyl-CoA with inversion of a-configuration upon decarboxylative condensation as found for fatty acid biosynthesis [15]. Meanwhile no deuterium label was detected at C-6, C-8, and C-12 positions, which bear the L-configured methyl substituents. Unfortunately, attempts to generate deuterated (2*R*)-methylmalonyl-CoA *in situ* by feeding cultures its direct precursor [2-^2^H_2_,2-^13^C]succinate (as a diethyl ester), were thwarted due to loss of the deuterium label by exchange processes. Thus the origin of the C-6, C-8, and C-12 positions with L-methyl substituents could not be clearly determined, as it could not be shown whether the extender units incorporated into these positions had arisen from (2*R*)-methylmalonyl-CoA with inversion of a-configuration, or from (2*S*)-methylmalonyl-CoA with subsequent a-epimerization (resulting in the loss of a deuterium) following an inverting condensation, or even if they had originated from (2*S*)-methylmalonyl-CoA (which may have lost its isotopic label through an exchange process) incorporated into the chain by a mechanism that retains a-configuration.

**Figure 7:**
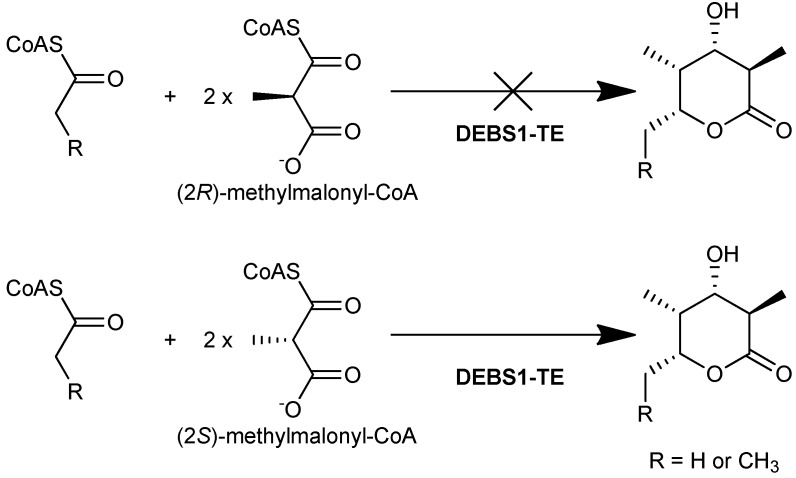
DEBS1-TE catalyses the formation of a triketide lactone product when using (2*S*)-methylmalonyl-CoA as a substrate, but does not form the product when (2*R*)-methylmalonyl-CoA is used [28].

In later studies, the isolation and characterization of the DEBS proteins [27] made it possible to elucidate the enantiomeric selectivity of the AT domains *in vitro*. Using radioactively labeled (2*R*)- and (2*S*)-[1-^14^C]methylmalonyl-CoA, it was demonstrated that DEBS1, DEBS2, and DEBS3 were radioactively labeled *via* acylation by (2*S*)-[1-^14^C]methylmalonyl-CoA, whereas no acylation of the proteins by (2*R*)-[1-^14^C]methylmalonyl-CoA was observed [28]. Limited proteolysis and subsequent labeling of the proteins showed that only fragments containing an AT domain carried the radioactive label, strongly suggesting that each of the AT domains of DEBS utilize solely the (2*S*)-methylmalonyl-CoA-derived extender unit. Unambiguous confirmation that this preference is exercised during chain extension came from *in vitro* studies of the engineered DEBS1-TE, which catalysed the formation of a triketide lactone product when supplied with (2*S*)-methylmalonyl-CoA along with NADPH and a suitable starter unit, but formed no product when (2*R*)-methylmalonyl-CoA was used instead (Figure 7). These results showed that the AT domains of both modules 1 and 2 of DEBS are selective for (2*S*)-methylmalonyl-CoA, even though the positions in the macrolide product corresponding to extension by those modules have opposite methyl configuration. Together these studies demonstrated that all six modules of DEBS employ (2*S*)-methylmalonyl-CoA for chain extension.

### 4.2. Stereochemistry of the KS-Catalysed Condensation

Following AT-catalysed attachment of an extender unit to the ACP, the KS domain condenses the growing polyketide chain to the ACP-bound extender unit. Considering that the KS-catalysed condensation in type I animal FAS occurs with inversion of configuration at the C-2 position of the extender unit, a logical assumption would follow that the KS domains of modular PKSs catalyse a condensation that likely also results in stereochemical inversion. A problem that arises from this assumption is that, although the AT domains of DEBS were all shown to select the (2*S*)- isomer of methylmalonyl-CoA, some of the methyl groups of the erythromycin product have L-configuration instead of the D-configuration that results from stereochemical inversion upon condensation of the (2*S*)-methylmalonyl extender unit [26,29]. To account for this, a number of possibilities were considered: either condensation can occur with inversion of stereochemistry at some centres, but retention at others; or epimerization can occur in some modules following condensation that results strictly in one of either inversion or retention; or some combination of scenarios in which condensation at various modules can occur with inversion or retention with or without epimerization.

**Figure 8:**
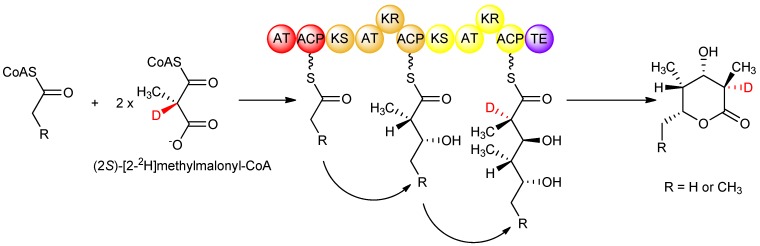
Isotopic labeling studies with DEBS1-TE showing that the D-configured methyl group arises from KS-catalysed incorporation of an extender unit from (2*S*)-methylmalonyl-CoA, while the L-configured methyl group arises from condensation with (2*S*)-methylmalonyl-CoA and subsequent epimerization [29].

Although isotopic labeling studies, in which deuterated precursors were fed to *S. erythraea*, showed that the D-configured methyl groups in erythromycin arose from a (2*S*)-methylmalonyl-CoA-derived extender unit that undergoes stereoinversion upon condensation, the origin of the L-configured methyl groups remained unclear from those studies. Knowing the strict stereospecificity of the AT domains of DEBS for (2*S*)-methylmalonyl-CoA, it was possible to investigate the stereochemistry of the subsequent condensation step catalysed by the KS domain, again by feeding isotopically labeled precursors *in vitro* to DEBS1-TE. In such a study [29], (2*RS*)-[2-^2^H]methylmalonyl-CoA was prepared and fed to DEBS1-TE, knowing that solely the (2*S*)- isomer is incorporated. Careful analysis showed only a single deuterium label in the product at the C-2 position (corresponding to module 2) bearing the D-configured methyl group, and no labeling at C-4 (corresponding to module 1) bearing the L-configured methyl group (Figure 8). The results together showed that in DEBS1-TE, decarboxylative condensation of (2*S*)-methylmalonyl-CoA in module 2 proceeds with inversion without cleavage of the C–H bond adjacent to the methyl group, while in contrast, the chain extension process in module 1 involves loss of the hydrogen attached to the α-position of the methylmalonyl-CoA precursor. Generation of the D-configured methyl group in module 2 without loss of hydrogen from the asymmetric centre of (2*S*)-methylmalonyl-CoA firmly established that the condensation occurs with inversion of configuration as observed in fatty acid biosynthesis, while in module 1, the loss of this key hydrogen from (2*S*)-methylmalonyl-CoA to produce the L-configured methyl centre implied that an additional obligatory epimerization step takes place in this module. These interpretations are supported by the earlier study in which isotopically labelled precursors were fed to erythromycin-producing cultures of *S. erythraea*, while the results of the *in vitro* experiments account for some of the ambiguous observations of that previous study [26]; validating the proposal that epimerization results in the L-configuration of the methyl groups. The nature of the epimerization reaction remains a matter of discussion. While some authors attribute the epimerase activity to the KS domain [29,30], others assign it to the downstream KR domain [31,32,33,34].

### 4.3. Stereospecificity of the KR-Catalysed Reduction of the b-Carbonyl Group

The KR domain, when present and active, catalyses stereospecific reduction of the 3-oxoacyl intermediate by transfer of the 4′-*pro*-*S* hydride from NADPH to the b-carbonyl, to give a hydroxyl of specific configuration [35]. In modules that utilize an extender unit with a substituent at the a-position, such as a 2-methyl group, if epimerization occurs on the a-position, the KR selects one epimer and catalyses stereospecific reduction of the a-branched b-ketoacyl intermediate, generating defined stereocentres at both a- and b-positions. In contrast to fatty acid biosynthesis, in which the KR always produces a hydroxyl in the D-configuration, the KR domains of modular PKSs are known to produce either D- or L-configured hydroxyl groups, depending on the module. The stereochemistry of ketoreduction was shown to be an intrinsic property of the KR domains when domain-swap experiments showed that when KR domains are placed into a hybrid PKS their native stereospecificity is carried through into their new context [36,37]. The stereospecificity of ketoreduction is thought to be determined by the binding orientation of the substrate, which is influenced by the identity of the amino acid residues on the unstructured loops flanking the active site [35,38]. KR stereospecificity can be assigned as either A-type, catalysing the formation of the an L-configured alcohol *via *a 3*S*-hydroxyl intermediate as seen in fatty acid b-oxidation, or B-type, catalysing formation the of a D-configured alcohol *via* a 3*R* hydroxyl intermediate as seen in fatty acid biosynthesis. The A-type and B-type KRs differ consistently in two regions of amino acid sequence. In the region designated ‘Motif I’ which includes residues 93 to 95 (numbering according to Caffrey [38]), the B-type KRs are typified by well conserved Leu93, Asp94, and Asp95 residues, which are absent in A-type KRs [38,39]. On the other hand, in ‘Motif II’ spanning residues 141 to 148, A-type KRs have a conserved Trp141, while B-type KRs typically have Pro144 and Asn148. X-ray crystal structures of both A-type [40] and B-type [31,32] recombinant KRs confirmed prior modeling and place these motifs in the active site. The solution of the crystal structure of TylKR_1_ (from the tylosin PKS) has led the authors of that study to propose further residues among A-type and B-type KRs which are posited to control selectivity of a-methyl configuration [32]. In the A-type KRs, the presence of His146 (keeping with the numbering of Caffrey for consistency) is proposed to distinguish KRs that are selective for the (2*S*)-epimer (the A2-type), resulting from a-methyl epimerization, from those which normally reduce the unepimerized (2*R*)- isomer (the A1-type). In B-type KRs, it has been proposed that a Pro151 distinguishes the (2*S*)- selective KRs (the B2-type) from those that reduce the (2*R*)- isomer (the B1-type). Even in non-functional KRs, it has been proposed that the replacement of a conserved Asn153 with smaller residues is indicative of selection of a (2*S*)-methyl-3-oxoacyl intermediate. Identification of these residues (Table 1) has allowed accurate prediction of the stereospecificity of KR domains and of the hydroxyl configuration of the products generated by their parent PKSs prior to empirical assignment of configuration [41,42,43]. 

**Table 1:** KR signature sequences.

KR type	Resulting intermediate	Conserved residues
A1-type	(2*R*,3*S*) 3-hydroxy-2-methylacyl	Trp141
A2-type	(2*S*,3*S*) 3-hydroxy-2-methylacyl	Trp141, His146
B1-type	(2*R*,3*R*) 3-hydroxy-2-methylacyl	Leu93, Asp94, Asp95, Pro144, Asn148
B2-type	(2*S*,3*R*) 3-hydroxy-2-methylacyl	Leu93, Asp94, Asp95, Pro144, Asn148, Pro151

This allows even prediction of the cryptic stereospecificity of KRs whose 3-hydroxyacyl products are subsequently dehydrated. In the overwhelming majority of cases of modules where ketoreduction is followed by either dehydration (typically resulting in a *trans* unsaturated intermediate) or both dehydration and enoylreduction, the KR has been predicted on the basis of sequence motifs to be B-type in stereospecificity, with few exceptions, the same stereospecificity observed in fatty acid biosynthesis [38]. The discovery of signature sequences within the KR domains of type I PKSs has also aided similar studies in understanding the stereospecificity of KRs in type II PKSs, of which little is known [44].

**Figure 9:**
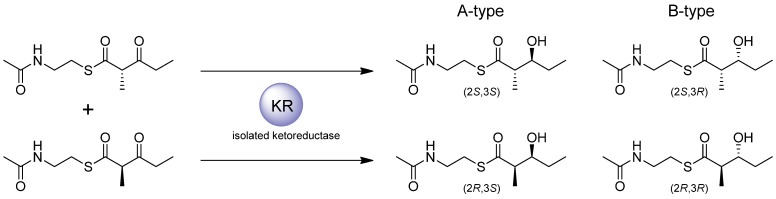
Reduction of surrogate substrates by recombinant KR domains isolated from their parent PKSs [35,45,46].

Cloning and expression of several recombinant type I KR domains as discrete enzymes allowed their intrinsic stereospecificity to be tested *in vitro* using a diketide *N*-acetylcysteamine (NAC) thioester as a surrogate substrate [35,45,46], and allowed the influence of conserved amino acids in Motifs I and II to be probed by mutagenesis. When (2*RS*)-methyl-3-oxopentanoyl-NAC was incubated with the purified KR domains derived from different PKS modules (Figure 9), the KR domains that normally encounter a-methyl diketide substrates resembling the surrogate substrate demonstrated exquisite stereospecificity at the b-hydroxyl and selectivity for epimers at the a-methyl, corresponding to the native stereochemistry of the enzymes with their natural substrates in the context of their parent PKSs. 

In contrast, when KRs that, in their native context, normally act upon substrates of greater chain-length were challenged with diketide substrates, the stereochemistry of the product is often scrambled with respect to the 3-hydroxy-2-methyl- intermediate produced in the natural PKS context, as was observed for EryKR_2_, EryKR_5_ and EryKR_6_ (from the erythromycin PKS) [35,47]. An exception to this is the A-type AmpKR_2_, the KR of the second extension module of the amphotericin cluster that normally acts upon a triketide, which was found to selectively reduce the (2*R*)- isomer of 2-methyl-3-oxopentanoyl-NAC to produce (2*R*,3*S*)-3-hydroxyl-2-methylpentanoyl-NAC, the expected configuration, in 92% diastereomeric excess [40]. Other *in vitro *studies using hybrid PKS modules, either as chimaeric constructs or reconstituted by mixing and matching of recombinant isolated PKS domains, showed that when EryKR_2_, EryKR_5_ or EryKR_6_, which normally act on tri- hexa- or heptaketide substrates, respectively, were presented with ACP-tethered triketide substrates generated *in situ*, these enzymes displayed strict A(1)-type stereospecificity as in their native context, while the same methodology was used to demonstrate that TylKR_1_ and TylKR_2_ exhibit their expected B(1)-type stereospecificity on ACP-bound triketide substrates [48,49].

In further *in vitro* studies carried out with the isolated EryKR_1_ and EryKR_2_ recombinant enzymes, site-directed mutagenesis was used to exchange the amino acid residues conserved in A-type KRs (such as EryKR_2_) for those typical of B-type (such as EryKR_1_) and *vice versa*. The stereospecificity of these mutants was determined using (2*RS*)-methyl-3-oxopentanoyl-NAC or -pantetheine thioesters as surrogate substrates. The parent EryKR_1_ reduced the surrogate substrates to produce almost exclusively the (2*S*,3*R*) 3-hydroxy-2-methylpentanoyl product. In Motif I of EryKR_1_, the mutations L93P, D94Q, and D95S resulted in a shift in the product stereochemistry to a 1:1 mixture of the (2*S*,3*S*) and (2*S*,3*R*) products. Mutations F141W and P144G in Motif II of EryKR_1_, resulted in a change in stereochemistry in which the (2*SR*)-methyl-3-oxopentanoyl substrate was reduced almost exclusively the (2*S*,3*S*) isomer. When all five mutations from Motif I and II flanking the EryKR1 active site were introduced, a complete switch in stereospecificity was observed with the (2*S*,3*S*) isomer being the sole product detected. Meanwhile, the parent EryKR_2_ displayed scrambled stereospecificity towards the unnatural (2*RS*)-methyl-3-oxopentanoyl, and when either the P93L, Q94D, Q95D mutations in Motif I, or the W141L, A144P, A148N mutations in Motif II, were introduced, the already scrambled stereospecificity shifted towards reduction of the surrogate substrate to a 1:1 mixture of (2S,3S) and (2S,3R) isomers. This was also observed when all six mutations flanking the EryKR_2_ active site were introduced. These results demonstrated that, by exchanging the key amino acids in A-type or B-type KRs using site-directed mutagenesis the stereochemistry at the resulting hydroxyl position could be shifted and in some cases completely switched. In other studies with isolated AmpKR_2_, analogous results have been obtained in switching the methyl configuration selected by KR domains by mutagenesis of key residues implicated in that process [40]. Later attempts to reintegrate mutant EryKR_1_ and EryKR_2_ domains with switched or altered stereospecificity into DEBS1-TE failed to produce any of the desired triketide compounds with altered stereochemistry when these mutant DEBS1-TE genes were introduced into an appropriate host organism, indicating that within the intact *in vivo* PKS system, there are factors that override the effect of mutations targeting the KR sequence motifs [50]. This may be attributed to poor processing by downstream enzymes with respect to the altered product [51,52,53], or the presence of discrete thioesterase enzymes in the *in vivo* system that may act to hydrolyse such (more slowly-processed) intermediates.

### 4.4. Stereochemistry of the DH-Catalysed Double-Bond Formation

Many complex polyketides originating from modular PKS-catalysed biosynthesis have carbon–carbon double bonds within their structures, and while a handful of these exist in the *cis* configuration, the large majority of unsaturated positions in polyketides are *trans*. In modules containing an active DH domain in addition to KR, the DH acts upon the b-hydroxyacyl intermediate, catalysing elimination of water to form an a,b-double bond. In the analogous reaction in the related FAS, the DH acts upon a (3*R*)-configured hydroxyacyl substrate to produce solely the *trans* (*E*) isomer of the unsaturated ∆^2^-acyl intermediate *via* the *syn* elimination of water [17].

**Figure 10:**
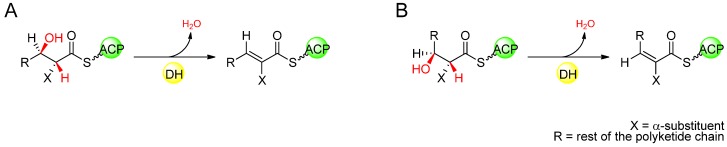
(**A**) DH-catalysed *syn* dehydration of a (3*S*)-hydroxyacyl intermediate, forming a *trans* ∆^2^-acyl intermediate. (**B**) DH-catalysed *syn* dehydration of a (3*R*)-hydroxyacyl intermediate, forming a *cis* ∆^2^-acyl intermediate.

In some examples of PKS modules that result in an unsaturated product with *trans* configuration, it has been demonstrated empirically that the DH domain acts specifically on a (3*R*)-hydroxyacyl intermediate, as in fatty acid biosynthesis. *In vitro* studies of a recombinant DH-containing PKS module from picromycin that yields a *trans*-configured double bond in the incorporated extender unit, proved that the KR that acts before the DH domain produced a (3*R*)-hydroxyacyl intermediate when the DH activity was absent [54]. Later studies were successful in isolating active recombinant DH domains from module 4 of the erythromycin PKS (DEBS), and module 2 of the nanchangmycin PKS, and showed that when assayed with ACP-tethered di- or triketides, EryDH_4_ and NanDH_2_ selected only the (3*R*)-hydroxyacyl substrates to produce a *trans* ∆^2^-acyl product by *syn* elimination of water [55,56]. Indeed, sequence analysis of PKSs having DHs in modules known to generate *trans* double-bonds predicts in most cases, that the KR in those modules is of the B-type, based on the identification of signature sequence motifs [38,39], generating an intermediate (3*R*) alcohol, suggesting that in general, *trans* double-bonds of polyketides arise from ketoreduction and subsequent DH-catalysed dehydration with the same stereochemistry as that of fatty acid biosynthesis. In the case of *cis* double bonds, though it has been shown that some of these arise from the action of exogenous enzymes not associated with the PKS [57,58,59], in other modular PKSs it has been proposed that the *cis* configuration arises from *syn* dehydration of a (3*S*)-hydroxyacyl intermediate by the DH domain (Figure 10) [39]. The implication of this hypothesis is that the double-bond geometry is determined by the stereochemistry of KR-catalysed ketoreduction rather than by the DH itself, with (3*R*)-hydroxyacyl intermediates generated by B-type ketoreduction undergoing *syn* dehydration to form a *trans* ∆^2^-acyl intermediate while (3*S*)-hydroxyacyl intermediates generated by A-type ketoreduction undergo *syn* dehydration to form a *cis* ∆^2^-acyl intermediate. Based on the predicted stereospecificity of KR domains by sequence analysis, this correlation often holds true, although known exceptions include the polyketides borrelidin [60], chivazol [61], difficidin [62], and mupirocin [63], where the KRs of the apparently *cis* double-bond-generating modules are predicted to be B-type.

X-ray crystal structures have recently been solved for the DH from the erythromycin PKS (DEBS) [64] and the DH domains from the curacin PKS, one of which appears to generate a *cis* double-bond [65]. These structures have shown that the DHs from modular PKSs show a very close similarity in overall fold and active site architecture to each other and to the related DH domains of animal FAS [66]. Catalysis has been posited to proceed *via* a His-Asp catalytic dyad mechanism in which the catalytic histidine abstracts the a-proton and the catalytic aspartatic acid donates a proton to the b-hydroxyl [64,65] with water then leaving, upon double-bond formation, from the same side of the acyl chain as proton abstraction, in a similar fashion to the reaction in fatty acid biosynthesis [67,68,69]. Although the solved structures of DH domains from modular PKSs contain no bound ligand, modeling suggests that the DH active site could in principle accommodate either (3*R*)- or (3*S*)-hydroxylacyl substrates to form either *trans* or *cis* unsaturated acyl products, respectively, by elimination of water [65].

Direct observation of the formation of *cis *double-bonds by DH domains within PKS modules has yet to be reported, but one study on the biosynthesis of phoslactomycins, a class of polyketides that contain multiple carbon–carbon double-bonds in the *cis* configuration, shows convincing evidence that the DH-containing first extension module of the phoslactomycin PKS produces a *cis* ∆^2^-acyl intermediate. In isotopic feeding studies, a mutant of phoslactomycin-producing *Streptomyces* sp HK803, in which the loading and first extension module of the phoslactomycin PKS were knocked out, was fed with [2-^13^C]-labeled *cis* and *trans* analogues of the ∆^2^-acyl intermediate [70]. Only the *cis* analogue was shown to be accepted by the downstream modules to restore phoslactomycin biosynthesis, strongly suggesting that the intermediate produced by the first module is the *cis* configured intermediate. Meanwhile it has been demonstrated that a *cis* double bond at another position in phoslactomycin is generated by a post-PKS tailoring enzyme [58].

An apparent exception to the observation that PKS modules that perform DH-catalysed formation of *cis* double-bonds contain A-type KRs is seen in the borrelidin PKS, in which modules 2 and 3 contain DH domains that catalyse the formation of the two carbon–carbon double bonds in the polyketide, one in the *trans* configuration and the other *cis*. Sequence analysis predicts that the KR domains in these modules both catalyse B-type ketoreduction, generating (3*R*) alcohols [71]. Recombinant expression of these isolated DH domains allowed their stereospecificity to be assayed *in vitro* using diketide surrogate substrates. Both DH domains had little or no activity towards (3*S*)-hydroxyacyl substrates and surprisingly were shown to dehydrate only the (3*R*) diketide alcohols to generate the *trans-*∆^2^-acyl products. In order to reconcile this with the fact that the double-bond formed by BorDH_3_ is *cis* in the borrelidin product but *trans* when assayed *in vitro* with surrogate substrates, several alternative explanations have been suggested: either the growing polyketide has an all-*trans* configuration, then subsequently undergoes catalysed *trans*-to-*cis* as proposed for the fungal hypothemycin [57]; or an exogenous enzyme directly catalyses formation of the *cis* double bond by binding to module 3 of the PKS, similar to the action of AveC during avermectin biosynthesis [59]; or the stereochemical outcome differs in the natural product during borrelidin biosynthesis as a result of the energetic differences between binding and catalysing dehydration of a diketide surrogate substrate *vs.* an ACP-bound heptaketide substrate.

### 4.5. Stereochemistry of the ER-Catalysed Reduction of the Double-Bond

**Figure 11:**
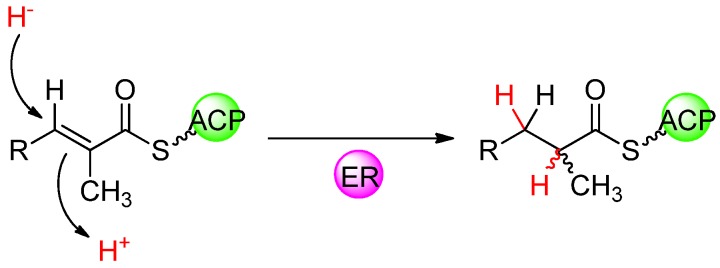
1,4-Nucleophilic addition of hydride ion, delivered from NADPH, to the unsaturated thioester followed by stereospecific protonation, which establishes the configuration of the methyl branch.

The ERs of modular PKSs and their counterparts in animal FASs belong to the medium-chain NAD(P)H-dependent dehydrogenase/reductase family (MDR) [72,73], and share a similar catalytic mechanism. In fully reducing modules, the ER domain reduces the 2-enoyl intermediate by catalysing the addition of a hydride from NADPH to the C-3 position of the enoyl substrate followed by protonation at the C-2 position (Figure 11). In modules that incorporate a branched extender unit, the configuration of the substituent at the C-2 position of the resulting intermediate depends upon the stereochemistry of enoylreduction. In principle it also depends on the geometry of the double bond, but the available evidence suggests that in the overwhelming majority of cases, the double bond is in the more stable *trans* arrangement [56], as in fatty acid biosynthesis. In contrast to the other individual domains of modular PKSs, the structure and function of the ER domain has been little-studied. Inspection of the outcome of ER-catalysed reduction in natural modular PKSs reveals that, in complex polyketide products, there are numerous examples of both L- and D-configurations at branched positions corresponding to C-2 in the ER-generated intermediate of the growing polyketide chain. This is reminiscent of the situation with KR domains, where it is possible to identify specific amino acid residues at the KR active site in modular PKSs that correlate with the stereospecificity of ketoreduction [38,39] which is either *R* or *S* depending on the face from which reduction occurs. The exact mechanism by which the ER controls the stereochemistry of reduction has yet to be fully elucidated, but recent research has suggested that, similar to the KRs, key amino acids influence the stereochemical outcome.

Multiple sequence alignment performed on ER domains from PKS modules in which a propionate unit (from methylmalonyl-CoA) becomes fully reduced and gives rise to a methyl branch of known configuration revealed a single amino acid (approximately 90 residues upstream of the NADPH-binding site) that shows excellent correlation with the configuration of the polyketide product [74]. In ER domains of modules that produce an L-configured methyl branch in the polyketide product (resulting from a 2*S*-methylacyl intermediate) this residue is systematically conserved as a tyrosine, while in the ER domains of modules that produce a D-configured methyl branch (resulting from a 2*R*-methylacyl intermediate) a valine (or occasionally alanine or phenylalanine) is found at this position. The position corresponds precisely to Tyr52 in the closely related *E. coli* quinone oxidoreductase and for ease of reference, this position in modular PKS ER domains is labeled 52′ with other residues within the ER domain numbered relative to it. This correlation was shown to hold for the methyl-branched lipids of mycobacteria as well as for complex polyketides. The PKS-biosynthesized waxy cell-wall lipids of many mycobacteria often have methyl branches that are of opposite configuration in closely related mycobacterial species, but otherwise identical. This difference can be traced to the ER domains of the PKSs that biosynthesize these lipids, which differ in the presence or absence of a tyrosine at 52′, but otherwise are very similar [74].

In domain-swap studies using hybrid PKSs derived from DEBS1-TE, it was demonstrated that replacement of the KR domain in the second module with either the full set of reducing domains (EryKR_4_, EryDH_4_, and EryER_4_) from module 4 of the erythromycin PKS (DEBS) or the full set of reducing domains (RapKR_13_, RapDH_13_, and RapER_13_) from module 13 of the rapamycin PKS (RAPS) resulted in the production of a 2-methyl triketide lactone that was fully reduced at the 3-position [37]. However, while the domains swapped from module 4 of the erythromycin PKS resulted in the production of a 2*S*-methyl product, those swapped from module 13 of the rapamycin PKS resulted in a 2*R*-methyl product. The results are in agreement with the fact that module 4 of DEBS incorporates an extender unit into erythromycin with an L-configured methyl substituent, consistent with the tyrosine at position 52′ of EryER_4_, whereas module 13 of RAPS incorporates an extender unit into rapamycin with a D-configured methyl group, consistent with the valine at position 52′ of RapER_13_. These domain-swapped hybrids provided a relatively simple system by which to study the specific role of key ER residues in stereospecificity using site-directed mutagenesis. In the hybrid PKS derived from DEBS1-TE, in which the KR of the second module was replaced with the KR, DH, and ER of DEBS module 4, the introduced EryER_4_ was mutated in position 52′, replacing tyrosine with valine (Y52′V), and the resulting mutant produced a triketide with 2*R*-methyl configuration, in contrast to the parent enzyme which produced a product with a 2*S*-configured methyl group [74]. However, in the hybrid PKS where the KR of the second module of DEBS1-TE was replaced with the KR, DH, and ER of RAPS module 13, an analogous mutation in RapER_13_ replacing valine at position 52′ for a tyrosine (V52′Y) resulted in no change in product configuration, yielding the same 2*R*-methyl triketide product as the parent. These results clearly demonstrated that the residue at position 52′ plays an important role in ER stereospecificity but that other residues must also be involved. This prompted a revised analysis of sequence alignments to identify other amino acid residues that correlate to the ER stereospecificity. In addition to the residue at position 52′, further analysis revealed three other positions that apparently correlate to stereospecificity [75]. 

**Figure 12:**
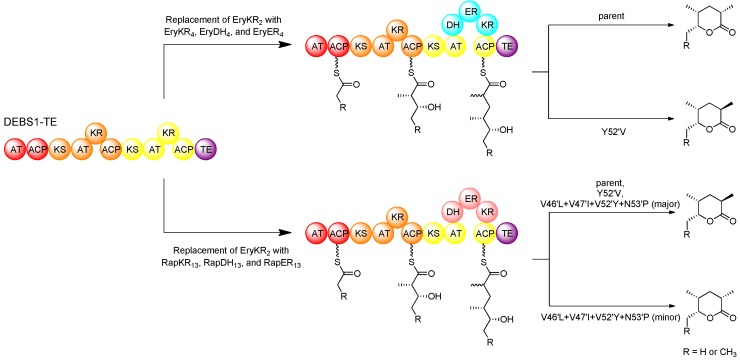
Domain swapping and mutagenesis with ERs in an intact PKS, resulting in altered product configuration [74,75].

Besides having a conserved Tyr52′, in the ERs that catalyse enoylreduction generating a 2*S*-configured intermediate (yielding an L-methyl group), residues 46′ and 47′ are typically conserved as leucine and isoleucine, respectively, and a proline at position 53′ is also fairly well conserved. On the other hand, in the ERs catalysing enoylreduction to a 2*R*-configured intermediate (yielding a D-methyl group) positions 46′ and 47′ are more commonly occupied by less bulky valine residues, and proline is not well conserved at position 53′. In the hybrid DEBS1-TE-derived PKS containing swapped KR, DH, and ER from RAPS module 13, several mutations (V46′L, V47′I, V52′Y, N53′P) were simultaneously introduced into these positions as well as the critical position 52′. The resulting mutant produced a small proportion of the 2*S*-configured triketide product along with the 2*R*-configured product, which is exclusively produced by the parent enzyme (Figure 12).

Together these studies revealed a handful of key residues that make up signature sequences (Table 2) that may be used to predict ER stereospecificity, and that may be targeted in rationally-guided attempts to engineer it, thereby generating novel polyketides of desired configuration. Such engineering of PKS ERs by rational design, however, is limited due to a lack of information on the structure or catalytic mechanism, although insights can be gathered from the ER domains of the closely related mammalian FASs for which X-ray crystal structures have been solved [66]. For example, in animal FAS, a key lysine residue of the ER is proposed to contribute to stereospecific catalysis of enoylreduction by donating a proton to C-2 at a specific face of the double-bond in the intermediate. Interestingly, mutation of the sequence homologous Lys236′ of RapER_13_ in the above-described hybrid PKS system resulted in a loss of stereospecificity at C-2 although it did not diminish polyketide production, suggesting perhaps that when this lysine is missing there is less control over the direction of protonation, and a solvent-derived proton might be delivered to either face of the intermediate [75].

**Table 2:** ER signature sequences.

Stereospecificity of ER	Conserved residues
(2*S*)-methyl	Leu46′, Ile47′, Tyr52′, Pro53′
(2*R*)-methyl	Val46′, Val47′, Val52′

## 5. Conclusions

The modular PKSs produce considerable structural diversity in their polyketide products, both in terms of functional groups and in stereochemistry arising from many stereogenic centres. In modular PKSs, the organization of modules and of the catalytic domains within them has long been recognized as the determinant of complex polyketide structure. In following the “co-linearity rule”, knowledge of the PKS organization may direct accurate predictions as to the structure of the polyketide product (and also the inverse of assigning a PKS to an identified product). More recently, aspects of stereochemistry in polyketide biosynthesis are becoming better understood. It is now possible to predict, based on the signature sequences within PKS domains, with relative accuracy, the configuration of many chiral centres within a polyketide product.

Along with new insights into the stereochemistry of polyketide biosynthesis comes hope that engineering the stereospecificity of PKSs shall become feasible for generating novel, desirable polyketide products. Indeed, early experiments have shown that engineered stereochemistry can be achieved in model PKS systems with some degree of success. Nevertheless, our current knowledge of the stereochemistry of complex polyketide biosynthesis is still maturing, as new developments shape our understanding of it.
